# Supplementary material for: Stacking Model‐Based Classifiers for Dealing With Multiple Sets of Noisy Labels
Source: Biom J. 2025 Mar 12;67(2):e70042. doi: 10.1002/bimj.70042 (PMC11898607; doi:10.1002/bimj.70042)
Supplement: Supplementary file 1 — Supporting Information [file BIMJ-67-e70042-s001.zip › density-based-ensemble-model-main/competitors_real_data/netlab-master/netlab-master/help/mdninit.htm]

Netlab Reference Manual mdninit


# mdninit

## Purpose

Initialise the weights in a Mixture Density Network.

## Synopsis

```
net = mdninit(net, prior)
net = mdninit(net, prior, t, options)
```

## Description

`net = mdninit(net, prior)` takes a Mixture Density Network
`net` and sets the weights and biases by sampling from a Gaussian
distribution. It calls `mlpinit` for the MLP component of `net`.

`net = mdninit(net, prior, t, options)` uses the target data `t` to
initialise the biases for the output units after initialising the
other weights as above. It calls `gmminit`, with `t` and `options`
as arguments, to obtain a model of the unconditional density of `t`. The
biases are then set so that `net` will output the values in the Gaussian
mixture model.

## See Also

`mdn`, `mlp`, `mlpinit`, `gmminit`

---

**Pages:**
Index


---

Copyright (c) Ian T Nabney (1996-9)

David J Evans (1998)
